# Personality functioning in anxiety disorders

**DOI:** 10.1186/s12888-018-1870-0

**Published:** 2018-09-14

**Authors:** Stephan Doering, Victor Blüml, Karoline Parth, Karin Feichtinger, Maria Gruber, Martin Aigner, Hemma Rössler-Schülein, Marion Freidl, Antonia Wininger

**Affiliations:** 1 Department of Psychoanalysis and Psychotherapy, Medical University of Vienna, Währinger Gürtel 18-20, A-1090 Wien, Austria; 2 Department of Psychology, Webster Vienna Private University, Wien, Austria; 3 Department of Social Psychiatry, Medical University of Vienna, Wien, Austria; 4 Department of Psychiatry, University Hospital Tulln, Tulln, Austria; 5 Vienna Psychoanalytic Society, Wien, Austria

**Keywords:** Personality functioning, Generalized anxiety disorder, Panic disorder, Phobia, Personality disorder

## Abstract

**Background:**

The Alternative DSM-5 Model for Personality Disorders as well as the upcoming IDC-11 have established a new focus on diagnosing personality disorders (PD): personality functioning. An impairment of self and interpersonal functioning in these models represents a general diagnostic criterion for a personality disorder. Little is known so far about the impairment of personality functioning in patients with other mental disorders than PD. This study aims to assess personality functioning in patients with anxiety disorders.

**Methods:**

Ninety-seven patients with the diagnosis of generalized anxiety disorder, panic disorder, or phobia, and 16 healthy control persons were diagnosed using the Structured Clinical Interview for DSM-IV (SCID-I and -II) and were assessed by means of the Structured Interview for Personality Organization (STIPO) to determine the level of personality functioning.

**Results:**

While all three patient groups showed significant impairment in personality functioning compared to the control group, no significant differences were observed between the different patient groups. In all three groups of anxiety disorders patients with comorbid PD showed significantly worse personality functioning than patients without. Patients without comorbid PD also yielded a significant impairment in their personality functioning when compared to the control group.

**Conclusions:**

Anxiety disorders are associated with a significant impairment in personality functioning, which is significantly increased by comorbid PD. There are no differences in terms of personality functioning between patients with different anxiety disorders.

## Background

Personality functioning has been introduced into the diagnosis of personality disorders (PD) by the two new classifications of mental disorders. Section III of the fifth edition of the Diagnostic and Statistical Manual of Mental Disorders (DSM-5) [1] contains an Alternative Model for Personality Disorders (AMPD; p. 761). In this model the general criterion A for PD is a “moderate or greater impairment in personality (self/interpersonal) functioning” (p. 761). This impairment is assessed by means of the DSM-5 Levels of Personality Functioning Scale (p.775) that comprises four dimensions: (1) self: identity, (2) self: self-direction, (3) interpersonal: empathy, and (4) interpersonal: intimacy. The so called beta draft of the upcoming International Classification of Diseases (ICD-11) contains a very similar description of “problems in functioning” as a general diagnostic criterion for PD, consisting of impaired functioning of “aspects of the self (e.g., identity, self-worth, accuracy of self-view, self-direction), and/or interpersonal dysfunction (e.g., ability to develop and maintain close and mutually satisfying relationships, ability to understand others’ perspectives and to manage conflict in relationships)” [2, 3].

Mental health and, as a consequence, psychosocial functioning are not only determined by the presence or absence of psychopathological symptoms, but also by basic functions of personality. In psychoanalytic theory, these functions are subsumed under the heading of psychic structure. Synonyms like personality structure, personality organization, or personality function are used frequently. Historically, Sigmund Freud was the first one who conceptualized psychic structure within his topographical model of conscious, preconscious, and unconscious psychic realms [4]. Later he developed his structural model by separating the ego, the id, and the super-ego as psychic structures [5]. Hartmann emphasized the relevance of ego functions for psychosocial adaptation as opposed to neurotic conflicts in the aetiology of mental disorders [6]. In his conceptual work Kernberg [7, 8] elaborated the impact of early experiences in relationships on the maturation of psychic structure or – in his words – personality organization. He was the first to define different levels of personality functioning and developed the *Structural Interview* as a clinical instrument for the assessment of personality organization [9]. The Structural Interview focuses key dimensions of personality function, i.e., identity integration, quality of object relations, defence mechanisms, superego integration (moral values), aggression, and reality testing. According to Kernberg, individuals with good (normal/neurotic) personality functioning show a consolidated identity, good quality of object relations, mature defence mechanisms, solid moral values and behaviour, secure reality testing, and are able to control their aggressive impulses. Patients with impaired personality function (Kernberg coined the term “borderline personality organization” for this group) suffer from identity diffusion, i.e., their internal images of the self and significant others are contradictory, superficial, and not integrated. Moreover, they are not able to maintain stable interpersonal relationships, are vulnerable to stress (employ primitive defence mechanisms), suffer from impaired impulse control, especially in terms of self-directed and other-directed aggression, and tend to have less stable moral values and behaviour. The most severely disturbed patients function on a psychotic level. In addition to an even worse personality functioning in all of the described domains they suffer from an impaired reality testing.

Since the Structural Interview is a clinical tool that does not allow for a reliable quantification of personality functioning, Kernberg and colleagues developed the Structured Interview for Personality organization (STIPO) [10] for research purposes. This 100-item interview addresses the domains mentioned above and results in a profile of personality functioning on seven dimensions and a total score on a six-point scale. The interview has been validated in its English original version and the German translation [11, 12]. The STIPO contains seven dimensions (see Methods section), the first two of which – identity and object relations – correspond to the two dimensions of the AMPD of DSM-5 and the personality functioning domains of the ICD-11 draft [1, 2].

During the last decade, a growing number of studies have been published that focus on personality functioning. It was demonstrated that patients with PD, especially borderline, show a worse personality functioning compared to patients with previously so called axis I disorders [11, 13], and that a higher symptom severity in borderline patients goes along with worse personality functioning [14]. However, very few studies have yet assessed personality functioning in disorders other than PD, e.g. in substance use disorders [15, 16].

The investigation of personality functioning in anxiety disorders seems of particular interest, since theoretical assumptions postulate different levels of functioning in different anxiety disorders. Eckhardt-Henn et al. (p.91) published a model that assigned the lowest level of personality functioning to patients with generalized anxiety disorder, a higher one to agoraphobic and panic disorder patients, and the highest one to individuals with specific phobias [17].

This study was undertaken to test the hypothesis that patients with different anxiety disorders reveal different levels of personality functioning, by assessing mental disorders including PD (formerly axis I and II disorders) according to DSM-IV [18] as well as personality functioning.

## Methods

### Study design

This study was approved by the ethics committee of the Medical University of Vienna, Austria on March 27, 2012 (IRB No. 1037/2012). After receiving detailed information about the study all subjects gave written informed consent. Patients and control subjects were diagnosed according to DSM-IV [18] by means of SCID-I and -II [19, 20] and underwent the Structured Interview for Personality Organization (STIPO) [10].

The patients were recruited between 2012 and 2017 at (1) the outpatient unit of the Department of Psychoanalysis and Psychotherapy, (2) the inpatient and outpatient units of the Department of Psychiatry and Psychotherapy, Division of Social Psychiatry of the Medical University of Vienna, (3) the Department of Psychiatry of the University Hospital Tulln, and (4) the outpatient clinic of the Vienna Psychoanalytic Society. In addition, a healthy control group from the community was recruited. In these individuals a screening with the Brief Symptom Inventory (BSI) [21] took place before the interviews (results are not reported here). All patients and control subjects received a compensation of € 50 for the participation in the study.

### Subjects

Inclusion criteria for all subjects were: (1) age ≥ 18 years and (2) sufficient knowledge of the German language. An additional inclusion criterion for patients was: (3) diagnosis of generalized anxiety disorder or panic disorder or specific phobia.

Exclusion criteria for all patients were: (1) organic brain disease, (2) mental disease with cognitive impairment (dementia, acute psychosis, severe depression), (3) substance dependence with acute intoxication, (4) comorbidity of two or all of the above mentioned anxiety disorders. Exclusion criteria for control subjects were: (5) a GSI (Global Severity Index) > 0.26 in the BSI screening and (6) any DSM-IV diagnosis according to SCID-I and -II.

### Instruments

#### Demographic data

The following demographic data were collected by means of a questionnaire: name, age, gender, marital status, educational status, and occupational status.

#### Structured clinical interview for DSM-IV (SCID-I and -II)

The SCID represents the American Psychiatric Association’s official instrument for the diagnosis of mental disorders according to DSM-IV. The structured interview contains questions addressing every single diagnostic criterion of the mental disorders of DSM-IV. The interview consists of two parts: SCID-I [20] for the assessment of all mental disorders except PD, which are evaluated by the SCID-II [19].

#### Structured interview for personality organization (STIPO)

The STIPO [10] was developed by Otto F. Kernberg and colleagues at the Cornell University New York. It represents the structured version of the Structural Interview that was developed by Kernberg in the 1980s [8, 9]. The interview contains 100 items that are addressed by one or more specific questions. The single-item rating is made by the interviewer on a three point scale with operationalized descriptions for each level: 0 = pathology absent, 1 = minor impairment, 2 = significant to severe impairment. The interview covers seven domains: (1) identity, (2) object relations, (3) primitive defences, (4) coping/rigidity, (5) aggression, (6) moral values, and (7) reality testing and perceptual distortions. Two different scoring systems can be used: (a) Guided by operationalized anchors each domain and subdomain is rated on a five-point scale with “1” standing for the absence of pathology to “5” indicating most severe impairment of personality functioning, an overall rating is generated from the ratings of the seven dimensions; (b) the arithmetic mean values are calculated for all dimensions and sub-dimensions from the single item scores (range: 0–2). Based on scoring system (a) six different levels of personality organization (i.e., personality functioning) are provided for the overall rating: (1) normal, (2) neurotic 1, (3) neurotic 2, (4) borderline 1, (5) borderline 2, and (6) borderline 3. Satisfactory reliability and validity of the English as well as of the German version of the instrument have been demonstrated [10, 11].

Both interviews were conducted by four well-trained postgraduate psychologists or medical doctors (A.F., K.F., K. P., M.G.) with proven reliability for the STIPO (ICC with expert ratings ≥0.7). One interviewer conducted both interviews in one and the same patient.

### Statistics

T-tests and one-way ANOVA were used for group comparisons of the level of personality functioning. Due to multiple testing in the group comparisons of the STIPO dimensions Bonferroni correction was employed and a level of significance of *p* < 0.006 was defined. Linear regression analyses were calculated for the evaluation of the effects of the type of anxiety disorder and comorbidity with personality disorder(s). IBM SPSS Statistics 24 (IBM Corporation, Armonk, New York, USA) was employed.

## Results

### Sample characteristics

In total, 97 patients and 16 healthy control subjects were included into the study. In the patient group, 22 were suffering from generalized anxiety disorder, 47 had panic disorder (with or without agoraphobia), and 22 phobias. Demographic data and diagnoses according to DSM-IV (American Psychiatric Association 1994) are given in Table 1. Regarding disorders other than PD (including the anxiety disorder) 26 (26.8%) had one diagnosis, 37 (38.1%) had two, 22 (22.7%) had three, 9 (9.3%) four, and 3 (3.1%) five or more diagnoses. Sixty-three (64.9%) were suffering from a comorbid personality disorder. Twenty-two (22.7%) had one personality disorder, 23 (23.7%) had two, 14 (14.4%) three, 2 (2.1%) four, and 2 (2.1%) five or more. There were no significant differences regarding comorbidity between the three groups of patients with anxiety disorders.

**Table 1:** Sample characteristics (*n* = 113)

	Phobias (*n* = 28)	Panic disorder (*n* = 47)	GAD (*n* = 22)	Controls (*n* = 16)
	Mean (s.d.)	Mean (s.d.)	Mean (s.d.)	Mean (s.d.)
Age (years)	30.96 (14.72) range: 18–73	35.26 (13.48) range: 19–74	36.55 (13.89) range: 19–70	31.88 (14.49) range: 20–60
	n (%)	n (%)	n (%)	n (%)
Gender				
Female	17 (60.7)	32 (68.1)	18 (81.8)	12 (75.0)
Male	11 (39.3)	15 (31.9)	4 (18.2)	4 (25.0)
Education				
No compulsory school	0 (0.0)	0 (0.0)	0 (0.0)	0 (0.0)
Compulsory school	5 (17.9)	11 (23.4)	5 (22.7)	0 (0.0)
Apprenticeship/ vocational school	9 (32.1)	11 (23.4)	6 (27.3)	3 (18.8)
High school	13 (46.4)	17 (36.2)	8 (36.4)	10 (62.5)
Academic	0 (0.0)	4 (8.5)	3 (13.6)	3 (18.8)
Other	1 (3.6)	3 (6.4)	0 (0.0)	0 (0.0)
Missing	0 (0.0)	1 (2.1)	0 (0.0)	0 (0.0)
Employment				
In occupational training	6 (21.4)	6 (12.8)	4 (18.2)	7 (43.8)
Unemployed	10 (35.7)	12 (25.5)	8 (36.4)	0 (0.0)
Part-time	1 (3.6)	8 (17.0)	4 (18.2)	5 (31.3)
Full-time	4 (14.3)	11 (23.4)	1 (4.5)	3 (18.8)
Homemaker	1 (3.6)	2 (4.3)	1 (4.5)	0 (0.0)
Retired	4 (14.3)	7 (14.9)	4 (18.2)	1 (6.3)
Missing	2 (7.1)	1 (2.1)	0 (0.0)	0 (0.0)
Family status				
Single	16 (57.1)	20 (42.6)	8 (36.4)	3 (18.8)
Unmarried with partner	9 (32.1)	12 (25.5)	5 (22.7)	9 (56.3)
Married	2 (7.1)	11 (23.4)	6 (27.3)	3 (18.8)
Divorced/ separated	1 (3.6)	2 (4.3)	3 (13.6)	1 (6.3)
Widowed	0 (0.0)	1 (2.1)	0 (0.0)	0 (0.0)
Missing values	0 (0.0)	1 (2.1)	0 (0.0)	0 (0.0)
DSM-IV diagnoses other than PD (anxiety disorders excluded)^a^			n	
Substance abuse disorders	4	15	2	0
Mood disorders	20	32	14	0
Brief psychotic disorder	1	2	0	0
Posttraumatic stress disorder	2	3	1	0
Obsessive-compulsive disorder	7	5	0	0
Somatoform disorders	3	2	0	0
Eating disorders	2	3	2	0
Adjustment disorders	1	0	0	0
DSM-IV PD Diagnoses^a^			n	
Paranoid	5	7	1	0
Schizoid	0	0	0	0
Schizotypal	0	0	0	0
Obsessive-compulsive	6	1	0	0
Histrionic	0	2	0	0
Dependent	4	2	2	0
Antisocial	1	0	0	0
Narcissistic	0	1	1	0
Avoidant	14	15	7	0
Borderline	10	9	4	0
Depressive	11	12	6	0
Passive-aggressive	0	2	1	0

*PD* personality disorder, *GAD* generalized anxiety disorder

^a^More than one diagnosis per patient included

#### Tests for normality of homogeneity of variance

Before using t-tests and ANOVA, tests for normality and homogeneity of variance were conducted. Neither the presence of PD nor the STIPO overall scores were normally distributed in the three subgroups of anxiety disorders. Shapiro-Wilk statistics for presence of PD in the three groups ranged from W = .508 to .628 with *p* < .001 and for the STIPO overall score W = .865 to .896 with *p* < .001, except for GAD with *p* = .011.

However, no significant inhomogeneity of variances occurred in the above mentioned subgroups. For this reason, it was decided to use the parametric tests the results of which are reported below.

#### Level of personality functioning in the groups of anxiety disorders

There were no significant differences between the different groups of patients with anxiety disorders regarding their level of personality functioning (see Table 2). The total mean score of the STIPO differed almost not at all. The mean values of 3.55 to 3.68 depict a moderate impairment of general personality functioning, or in terms of the STIPO model an organization between lower neurotic and higher borderline functioning. Compared to the healthy control group with a mean STIPO score of 1.50, all three patient groups showed significant impairment. The distribution of the level of personality organization in the four groups is depicted in Table 3. Post-hoc comparisons (least significant difference; Bonferroni corrected level of significance *p* < .004) revealed highly significant differences between controls and all three groups of anxiety disorders (*p* < .001) and non-significant results between the groups of anxiety disorders (*p* = .625 to .838).

**Table 2:** Personality organization in the different groups of anxiety disorders (*n* = 113)

	Phobias (*n* = 28)	Panic disorder (*n* = 47)	GAD (*n* = 22)	Controls (*n* = 16)	ANOVA (including controls)		ANOVA (without controls)			
	Mean (s.d.)	Mean (s.d.)	Mean (s.d.)	Mean (s.d.)	F	df	*p* ^a^	F	df	*p* ^a^
STIPO dimensions										
Identity	2.79 (±0.83)	2.62 (±0.85)	2.64 (±0.95)	1.25 (±0.45)	13.88	3	<.001	0.35	3	.70
Object relations	2.68 (±0.86)	2.51 (±0.98)	2.45 (±0.91)	1.44 (±0.63)	7.34	3	<.001	0.43	3	.66
Primitve defenses	2.86 (±0.85)	2.70 (±0.98)	2.77 (±0.92)	1.19 (±0.40)	15.01	3	<.001	0.27	3	.78
Coping/ rigidity	3.43 (±0.69)	3.04 (±0.81)	3.36 (±0.79)	1.56 (±0.51)	25.03	3	<.001	2.65	3	.08
Aggression	1.89 (±0.88)	2.09 (±0.91)	2.05 (±1.09)	1.00 (±0.00)	6.53	3	<.001	0.38	3	.69
Moral values	1.61 (±0.69)	1.60 (±0.71)	1.68 (±0.95)	1.19 (±0.54)	1.64	3	.19	0.10	3	.91
Reality testing and perceptual distortions	2.04 (±1.04)	2.26 (±1.01)	1.86(±1.04)	1.13 (±0.34)	5.66	3	.001	1.18	3	.31
Total score	3.68 (±0.95)	3.60 (±1.04)	3.55 (0.96)	1.50 (±0.63)	22.52	3	<.001	0.12	3	.89

*GAD* generalized anxiety disorder, *ANOVA* analysis of variance, *STIPO* Structured Interview for Personality Organization

^a^Bonferroni corrected, level of significance *p* < 0.006

**Table 3:** Personality organization in the different groups of anxiety disorders (*n* = 113)

	Phobias (*n* = 28)	Panic disorder (*n* = 47)	GAD (*n* = 22)	Controls (*n* = 16)
	n (%)	n (%)	n (%)	n (%)
STIPO level of personality organization				
1 – normal	0 (0.0)	0 (0.0)	0 (0.0)	9 (56.3)
2 – neurotic 1	2 (7.1)	7 (14.9)	2 (9.1)	6 (37.5)
3 – neurotic 2	11 (39.3)	17 (36.2)	10 (45.5)	1 (6.3)
4 – borderline 1	10 (35.7)	11 (23.4)	7 (31.8)	0 (0.0)
5 – borderline 2	4 (14.3)	12 (25.5)	2 (9.1)	0 (0.0)
6 – borderline 3	1 (3.6)	0 (0.0)	1 (4.5)	0 (0.0)

*GAD* generalized anxiety disorder, *STIPO* Structured Interview for Personality Organization

In correspondence to the overall score, the seven dimensions of the STIPO did not show relevant differences between the groups of anxiety disorders. Within all groups the best personality functioning occurred in the domain of moral values, which indicates a low level of antisocial tendencies. Also the aggression and reality testing domains yielded slightly higher levels of personality functioning than the remaining scales.

#### Comorbid personality disorders and level of personality functioning

In view of the high number of comorbid personality disorder diagnoses in the sample it was tested, whether a PD is of more relevance for personality functioning than the type of anxiety disorder.

A linear regression analysis was conducted to further explore the influence of anxiety disorders as well as comorbid PD on personality functioning. It turned out that only the presence of a PD was significantly associated to with personality functioning, but not any of the three anxiety disorders - a comorbid PD impaired personality functioning (*T* = 8.121, *p* < .001).

However, an ANOVA excluding all patients with PD still yielded a significantly worse personality functioning in patients compared to controls (F = 14.020, df = 3, *p* < .001). Patients without comorbid PD showed a moderate impairment of personality function with a mean STIPO score of around 3, which indicates a lower neurotic level, whereas patients with comorbid PD revealed a mean STIPO score around 4, which stands for a higher borderline organization (see Table 4).

**Table 4:** Personality organization (STIPO overall scores) in the different groups of anxiety disorders with and without comorbid personality disorders (*n* = 97)

	STIPO level of personality organization	ANOVA		
	Mean (s.d.)	F	df	*p*
Anxiety disorder				
Phobias without PD (*n* = 6)	2.83 (±0.75)	−2.76	26	.011
Phobias with PD (*n* = 22)	3.91 (±0.87)			
Panic disorder without PD (*n* = 19)	2.84 (±0.83)	−5.11	45	<.001
Panic disorder with PD (*n* = 28)	4.11 (±0.83)			
GAD without PD (*n* = 9)	3.11 (±0.60)	−1.86	20	.077
GAD with PD (*n* = 13)	3.85 (±1.07)			
All patients without PD (*n* = 34)	2.91 (±0.75)	−5.97	95	<.001
All patients with PD (*n* = 63)	3.98 (±0.89)			

*PD* comorbid personality disorder, *GAD* generalized anxiety disorder, *ANOVA* Analysis of Variance, *STIPO* Structured Interview for Personality Organization

Finally, when all 113 subjects were included in a correlation analysis of number of PDs diagnosed and the STIPO overall score a highly significant correlation emerged (*r* = 0.596, *p* < .01).

## Discussion

Our data demonstrate that anxiety disorders are associated with an impaired personality functioning, which is moderate when no comorbid personality disorder is present and more severe in case of comorbid PD. There are no differences in personality functioning between phobias, GAD, and panic disorder, either with or without comorbid PD.

The hypothesis derived from psychoanalytic theory that GAD patients are characterized by a worse personality functioning followed by panic disorder patients and phobia patients with the best functioning could therefore not be confirmed. In contrast, it seems that anxiety disorders can occur on all levels of personality organization from mild impairment (“high neurotic level”) to the lowest level (“low borderline level”) (see Table 3). It is possible that in first level care settings patients with anxiety disorders with higher personality function can be found compared to patients in our study of secondary and tertiary care settings. In a Swedish study by Sundquist et al. about half of the patients with PD were only present in the primary care setting and never showed up in secondary or tertiary care settings [22].

The pattern of personality functioning within the seven domains of the STIPO seems to reflect the comparably high ratio of avoidant and depressive PD and the lower prevalence of Cluster A and B PD in the sample (see Table 1). One might assume that Cluster C PD show a higher impairment in the realms of relational functioning as well as coping with and defending against stress. However, the validity study of the German version of the STIPO [10] revealed a similar pattern in a sample with a much higher ratio of Cluster A and B PD. Thus, personality functioning appears to be rather independent from the symptoms and characteristics of specific PDs.

A very high comorbidity between anxiety disorders and PDs has previously been shown. Grant et al. [23] reported from the National epidemiologic survey on alcohol and related conditions (NESARC) that in 41.8% of patients with any anxiety disorder a comorbid personality disorder is present. In treatment seeking patients with anxiety disorders this number rises to 59.6%. Thus, our finding of 64.9% of comorbid PDs in a population from psychiatric in- and outpatient units is in line with this earlier finding. It can be assumed that a study recruiting subjects with anxiety disorders outside the mental health care system would have found somewhat lower rates of comorbid PDs and, thus, a slightly better personality functioning.

Our study adds to these previous findings the result that even in absence of a personality disorder patients with an anxiety disorder show significant impairment in their personality functioning. As a consequence, the treatment of patients with anxiety disorders should take into consideration the presence of personality problems. On the one hand, a diagnosis of personality functioning as well as PD should be mandatory in every patient. The newly developed instruments for the assessment of the levels of personality functioning according to DSM-5 serve this purpose, e.g., the self-report form of the DSM-5 Level of Personality Functioning Scale [24, 25]. Moreover, well-established questionnaires and interviews exist that can be employed. For example, the Inventory of Personality Organization (IPO) [26, 27], the Structured Interview for Personality Organization (STIPO) [10, 11], and the Operationalized Psychodynamic Diagnosis (OPD-2) [28]. It has recently been shown that the DSM-5 levels of personality functioning can be reliably assessed by untrained raters from audio-recorded STIPO interviews [29] as well as OPD-2 interviews [30]. For a general assessment of personality function and screening purposes, the DSM-5 LPFS or questionnaires can be used, while the multidimensional and more extensive interviews like the STIPO or OPD-2 yield a much more comprehensive assessment of the different domains of personality functioning, which is highly valuable for clinical treatment planning as well as for detailed research questions.

On the other hand many treatment failures in patients with anxiety disorders might be attributable to impaired personality functioning and comorbid PD [31–33]. This is of high clinical relevance, since it is known, that treatment response rates are low, e.g. 48% in generalized anxiety disorder as reported by Hunot et al. [34] in their Cochrane review. If one assumes, that most of the randomized-controlled treatment studies exclude patients with comorbid PD, the relevance of sub-threshold/ mild impairment of personality functioning might be of even greater importance for treatment outcome. Personality functioning can be improved by psychotherapy, which has been shown by Doering et al. [35], but it will need specialized treatment approaches that focus not only on symptoms of the anxiety disorder, but also on specific domains of the patient’s personality [36]. If we follow the presumption that personality pathology complicates the treatment of anxiety disorders and has to be addressed before sustainable symptom remission can occur, specific treatments should be offered to patients with both, anxiety disorder and impaired personality functioning. Treatments that have demonstrated their efficacy in improving personality functioning are Transference-focused Psychotherapy (TFP) [35, 37–39] and - with some limitations due to the lack of specific outcome criteria in the conducted RCTs - Schema-focused Psychotherapy (SFT) [40, 41], Mentalization-based Treatment (MBT) [42–44], and Dialectical Behavior Therapy (DBT) [45, 46].

Limitations of this study can be found in the relatively low sample size in two of the three groups of patients with anxiety disorders, and in the recruitment at psychiatric hospital units. This reduces the generalizability of the absolute numbers regarding personality functioning in anxiety disorders, which probably is somewhat better in the general population than in the treatment seeking sub-population. However, the result that all anxiety disorders occur on almost all levels of personality functioning can be regarded as mainly independent from the recruitment bias. In future studies with larger sample sizes it will be interesting to evaluate the specific influence of cluster B vs. cluster A or C PDs, or even of specific PDs like borderline.

## Conclusions

An impairment of personality functioning is highly frequent in all anxiety disorders and has to be taken into consideration for diagnosis and treatment of patients with anxiety disorders. There are no differences between the anxiety disorders with regard to their personality functioning, and comorbid PD further impair personality functioning.

## Abbreviations

AMPD: Alternative Model for Personality Disorders
ANOVA: Analysis of Variance
BSI: Brief Symptom Inventory
DBT: Dialectical Behavior Therapy
DSM-5: Diagnostic and Statistical Manual of Mental Disorders – Fifth Edition
DSM-IV: Diagnostic and Statistical Manual of Mental Disorders – Fourth Edition
GAD: Generalized Anxiety Disorder
GSI: Global Severity Index
ICC: Intraclass Correlation
ICD-11: International Classification of Mental and Behavioral Disorders, 11th Revision
IPO: Inventory of Personality Organization
LPFS: Levels of Personality Functioning Scale
MBT: Mentalization-based Treatment
NESARC: National Epidemiologic Survey on Alcohol and Related Conditions
OPD-2: Operationalized Psychodynamic Diagnosis, Second Edition
PD: Personality Disorder
SCID-I: Structured Clinical Interview for DSM-IV, Axis I
SCID-II: Structured Clinical Interview for DSM-IV, Axis II
SFT: Schema-focused Psychotherapy
STIPO: Structured Interview of Personality Organization
TFP: Transference-focused Psychotherapy
